# Plasma Exchange in the Setting of Acute Ibuprofen Poisoning: A Case and Review of the Literature

**DOI:** 10.3390/reports9030228

**Published:** 2026-07-16

**Authors:** Raeshun T. Glover, Robert W. Maitta, Yi-Yuan Zhou

**Affiliations:** Department of Pathology, University Hospitals Cleveland Medical Center, Case Western Reserve University, Cleveland, OH 44106, USA; robert.maitta@uhhospitals.org (R.W.M.); yiyuan.zhou@uhhospitals.org (Y.-Y.Z.)

**Keywords:** ibuprofen toxicity, therapeutic plasma exchange (TPE), continuous renal replacement therapy (CRRT), acute kidney injury, extracorporeal toxin removal

## Abstract

**Background and Clinical Significance**: Extracorporeal therapies such as hemodialysis, continuous renal replacement therapy (CRRT) or therapeutic plasma exchange (TPE) are mainstay treatments for acute toxins, venoms and poisonings. Ibuprofen is a common drug implicated in acute toxicity, with a major complication being acute kidney injury (AKI). As such, these patients often receive hemodialysis or CRRT, which do not remove substances with high protein binding (>80%) and low volumes of distribution (Vd) (<0.2 L/kg). The pharmacokinetics of ibuprofen have these characteristics. While hemodialysis or CRRT can clear the accumulated toxic metabolites, they do not remove the ibuprofen that causes renal injury. In contrast, TPE can remove protein-bound drugs such as ibuprofen. **Case Presentation**: We describe the case of a previously healthy young male who presented with AKI due to ibuprofen toxicity requiring concurrent continuous renal replacement therapy and plasma exchange. **Conclusions**: TPE in conjunction with hemodialysis or CRRT is an effective treatment modality for ibuprofen toxicity.

## 1. Introduction and Clinical Significance

Extracorporeal therapies are an emerging approach for treating acute toxin exposure/poisonings. A major extracorporeal therapy that has been used with good clinical efficacy is hemodialysis. Unfortunately, this modality has been employed primarily to treat acute exposure to a limited selection of acute toxins such as alcohol, lithium and salicylates. This is because hemodialysis does not effectively remove heavily protein-bound toxins (>80% protein binding) [1]. By contrast, another extracorporeal modality, therapeutic plasma exchange (TPE), removes acute toxins specifically when they are highly protein-bound, providing an alternative treatment modality [2].

The American Society for Apheresis (ASFA) categorizes apheresis procedures according to the quality of evidence for individual indications. TPE for acute toxins, venoms and poisons is currently classified in the ASFA 2023 guidelines as Category III, grade 2C [2]. This denotes that the optimum role for TPE has not been established and with weak quality evidence supporting TPE use for this indication. Given the various pharmacokinetics of different toxins, and the fact that TPE works best with substances with a low volume of distribution (Vd) (<0.2 L/kg) and high protein binding (>80%), the effectiveness of TPE would be dependent on a per-toxin basis [3]. As such, case studies/series on individual toxins are necessary to determine the clinical effectiveness of TPE within this broad indication. There are few case reports on TPE use in the setting of acute ibuprofen poisoning [4,5,6]. Often in these cases, TPE is used concomitantly with continuous renal replacement therapy (CRRT) or hemodialysis. Herein we present a case of TPE used in conjunction with CRRT for significant acute ibuprofen poisoning.

## 2. Case Presentation

The patient was a young male with no significant past medical history who presented to the emergency department reportedly within 6 h of ingestion of multiple bottles containing ibuprofen of unspecified size and dosage. He presented unresponsive and was admitted requiring intubation. Analysis of urine and blood for common toxins was performed (Table 1). The urine drug screen was positive for cannabinoids. An acute toxicity panel was performed in-house and was negative for acetaminophen, salicylate and ethanol. An ibuprofen level was sent out to a reference lab but given the clinical history, clinical presentation and the exclusion of other drugs via the urine testing and acute toxicity panel, a presumptive diagnosis of acute ibuprofen poisoning was made. Throughout the day, he developed worsening lactic acidosis, increasing serum creatinine levels and worsening hypotension. Initial care was supportive with administration of sodium bicarbonate, calcium gluconate, vasopressor (norepinephrine) and fluids (5% dextrose and 0.9% sodium chloride). The patient’s circulatory shock was refractory to vasopressors, requiring increasing epinephrine, vasopressin and norepinephrine drips. Overall, the patient had severe acute kidney injury (AKI), sinus tachycardia, toxic encephalopathy and refractory circulatory shock as a sequela of suspected intentional ibuprofen poisoning. Extra-corporeal membrane oxygenation (ECMO) was initiated due to hemodynamic instability refractory to increasing vasopressors. The patient was initiated on heparin according to institutional ECMO protocol, which includes a loading dose of 20 units/kg/h, and rechecking anti-Xa levels every four hours or after rate change for a target of 0.3 IU/mL. CRRT was initiated for the patient’s severe AKI. Regional CRRT anticoagulation included anticoagulant citrate dextrose solution A (ACD-A) at a rate of 300 mL/h. The initial calcium chloride (8 g in 0.9% sodium chloride, 1000 mL) rate was 90 mL/h and titrated based on ionized calcium every 2 h (10 mL/h adjustments based on level). Given that ibuprofen is not effectively removed via hemodialysis or CRRT, transfusion medicine was consulted to initiate TPE for this patient.

Benefits (potential removal of ibuprofen and improvement of symptoms of toxicity) vs risks (citrate toxicity, bleeding risk, allergic transfusion reaction, transfusion-related acute lung injury, transfusion-transmitted infection, etc.) were discussed with the consulting team and healthcare proxy, and the decision was made to proceed with emergent TPE. CRRT was paused during TPE. A 1.0× plasma volume exchange (Height: 190.5 cm; Weight: 83.5 kg; plasma volume: 3600 mL) was performed via an IVJ dialysis catheter using a 50:50 ratio of 5% albumin to ABO-type specific plasma on the Spectra Optia Apheresis System (Terumo BCT, Lakewood, CO, USA), following the manufacturer’s specifications and our institutional protocols. Citrate Dextrose-A was used as anticoagulant solution. The patient was already placed on a high-dose calcium IV drip by the intensive care team as mentioned previously. The procedure had a runtime of 183 min and was performed without complication. A post-TPE ibuprofen level was drawn, and the patient was monitored clinically for improvement, with the transfusion medicine team remaining on consultation for determination of whether further TPE procedures were indicated.

**Figure 1:**
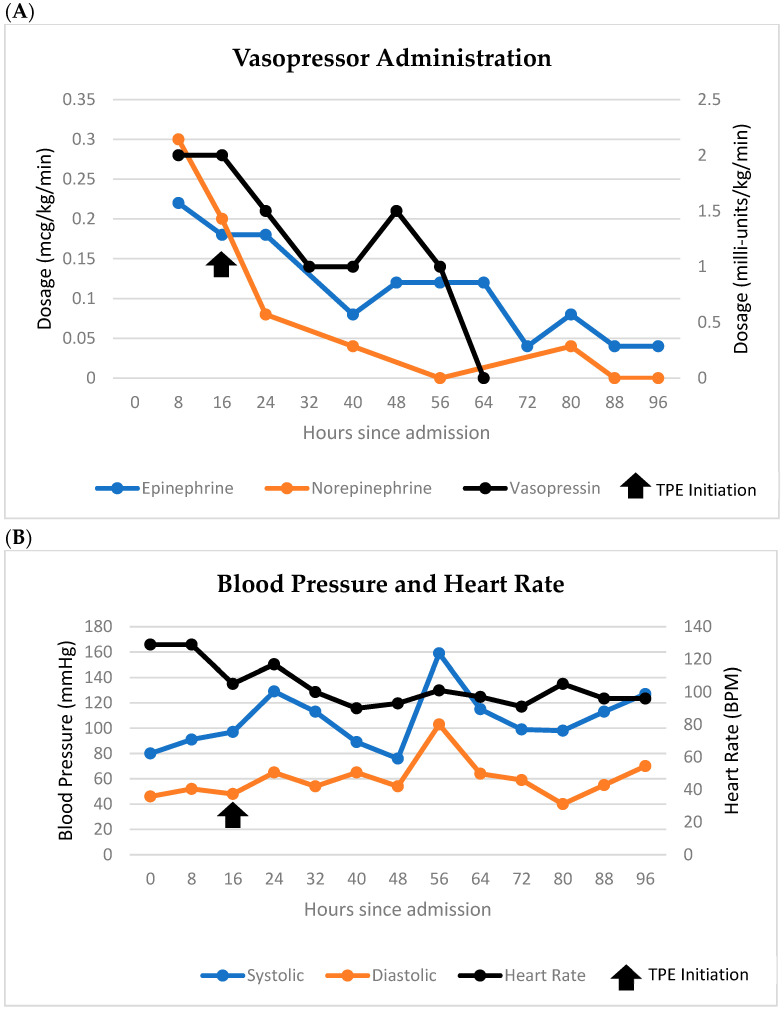

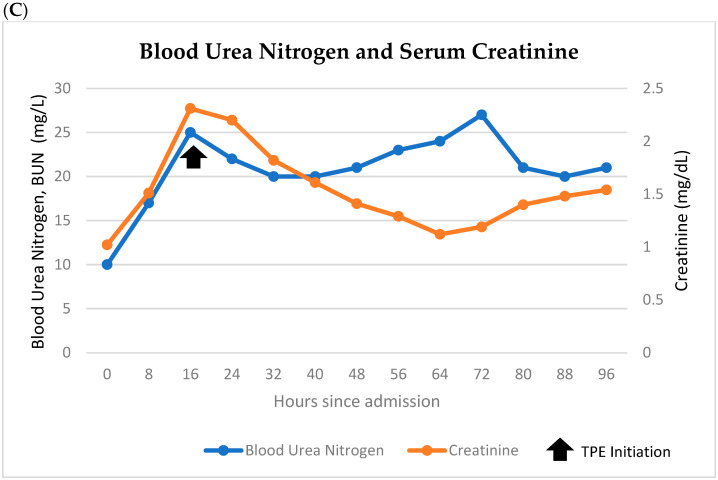
Hemodynamic and renal parameters before and after TPE. (**A**) Vasopressor requirements. (**B**) Blood pressure and heart rate. (**C**) Blood urea nitrogen and serum creatinine. ECMO and CRRT were initiated at approximately 15 h post-admission. TPE was performed at approximately 16 h post-admission.

In regard to the circulatory shock, the patient improved in the 72 h post-TPE as demonstrated by decreasing vasopressor requirements and normalization of blood pressure and heart rate (Figure 1A,B). Creatinine and BUN levels after CRRT initiation and TPE initially demonstrated a downtrend with a slight uptrend on day 3 post-admission (Figure 1C). Within 24 h post-TPE, the patient was able to start the weaning process for vasopressors, bicarbonate, and insulin infusion. Given the evident early clinical improvement on day 2, a second TPE was deferred. The patient was removed from ECMO 3 days after TPE, and CRRT was discontinued 5 days later. The remainder of his hospital course from the AKI perspective was uncomplicated, with slow recovery of his renal function as shown by a downtrend in cystatin C and creatinine levels in the setting of persistent hypertension.

Of note, the patient had two bleeding complications. The patient experienced a gastrointestinal (GI) bleed two days after TPE, requiring gastrotomy and clot removal. Esophagogastroduodenoscopy showed that the bleed was secondary to severe acute gastritis, secondary to acute ibuprofen poisoning. The patient also developed pulmonary hemorrhage on day 6, which delayed respiratory recovery. Ventilation was continued until extubation on day 11. While the post-procedure fibrinogen was decreased, the patient had a low-normal level prior to the procedure, and it would be expected to increase over the next 48 h as the patient’s liver labs showed no significant acute hepatic injury. On post-procedure day 2, when the GI bleed occurred, fibrinogen level was appropriate for hemostasis at 397 mg/dL. While the procedure removed some heparin from the patient (38% reduction), the overall coagulation labs showed persistent anticoagulation with therapeutic unfractionated heparin (Table 1). Overall, we deem these bleeding episodes unrelated to TPE as we opted to use a 50:50 albumin to plasma ratio to maintain the patient’s coagulation labs approximately at pre-procedure values consistent with their baseline on heparin anticoagulation for ECMO.

A send-out ibuprofen level resulted approximately a week after TPE. Ibuprofen poisoning was confirmed with a level of 620 μg/mL. Thus, TPE reduced the serum ibuprofen level by approximately 68% from 620 μg/mL to 200 μg/mL.

## 3. Discussion

Our case showed an approximately 68% reduction in serum ibuprofen levels after a single 1.0× plasma volume TPE with a 50:50 albumin to plasma ratio. Additionally, the patient experienced significant clinical improvement in the post-exchange clinical course, shown by decreasing vasopressor support and improving blood pressure and heart rate.

The Vd (0.1–0.2 L/kg) and protein binding (>98%) of ibuprofen theoretically make it a reliable substance to be removed by TPE, with a theoretical maximum removal of 65–70% [3,7]. Despite this, multiple studies reported suboptimal ibuprofen removal in a single TPE session or required multiple TPE sessions to reach the theoretical maximum removal [4,5,6]. Extensive reviews of the published literature revealed a total of three cases of TPE utilized in acute ibuprofen poisoning. Of these three cases, two were meeting abstracts.

Geith et al. reported a case of clinical improvement in the setting of AKI secondary to acute ibuprofen poisoning treated by performing TPE (100% plasma; unspecified plasma volumes exchanged) followed by serial hemodialysis that decreased vasopressor requirement post-procedure, like our case [4]. They reported an approximately 30% reduction in the ibuprofen level from 275 μg/mL to 180 μg/mL following a single TPE procedure [4]. In this case, a pharmacokinetic profile was also created utilizing serial pre- and post-TPE ibuprofen levels. The pharmacokinetic profile demonstrated that while there was removal of ibuprofen after TPE, a rebound occurred at approximately 10 h post-procedure with a subsequent decrease at 20 h post-procedure. This was likely due to re-equalization from extravascular spaces. Manning et al. reported findings similar to our case in a patient with acute ibuprofen poisoning treated with TPE and CRRT [5]. A TPE (unspecified replacement fluid; 1.0× plasma volumes exchanged) was performed, which showed a 35.7% reduction in serum ibuprofen from 420 μg/mL to 270 μg/mL. They weaned vasopressors within 24 h post-TPE and discontinued CRRT 48 h post-TPE. This case also had GI bleeding as an associated condition as well. Finally, Vansickle demonstrated an approximately 30% reduction in ibuprofen level from 330 μg/mL to 200 μg/mL following one TPE (unspecified replacement fluid and plasma volumes exchanged) [6]. They subsequently performed serial procedures every 12 h for a total of four procedures and did not reach a greater than 68% reduction from the initial serum ibuprofen level until the fourth procedure.

Overall, this series of cases, including our case, has shown that TPE with hemodialysis or CRRT has led to clinical improvement in the days after the procedure; specifically, with decreased vasopressor needs. In contrast to prior published cases where the percent reduction in ibuprofen ranged from approximately 30–35%, our case showed approximately a 68% reduction in serum ibuprofen levels after a single procedure.

We attribute the effectiveness of this procedure to prompt initiation of TPE in relation to the consumption of ibuprofen. Firstly, the effectiveness of ibuprofen removal is greatest at or near the attainment of peak plasma concentration. Secondly, there is a narrow window during which ibuprofen will peak and ultimately be metabolized by the liver. Ibuprofen peaks within up to 2 h after ingestion and has a half-life ranging from 1 to 3 h [7]. The expected clearance of ibuprofen is approximately five half-lives or 5–15 h. This is highlighted by the fact that in Geith et al., the plasma ibuprofen level decreased 50% spontaneously from the time of admission until the start of TPE, which was a period of approximately 5 h [4]. The decreased pre-procedure ibuprofen level could explain the lower efficiency of their TPE procedure. Given this information, this procedure may best be performed within 12–15 h of ingestion. Given the demonstration from Geith et al. that rebound can occur, additional procedures may be indicated [4]. If rapid (<24 h) ibuprofen levels are available, a post-procedure ibuprofen level should be obtained for determination of further TPE procedures. As ibuprofen level is typically a reference lab test, many institutions may not have rapid testing and may receive results later (>24 h). In these cases, determination of further procedures can be assessed by clinical improvement (vasopressor requirements, blood pressure, heart rate) over the next 24 h post-TPE. The post-procedure ibuprofen level can be used for quality assurance to maintain consistent ibuprofen removal metrics.

While this case demonstrates effective removal of ibuprofen by TPE, causal links between ibuprofen removal and clinical improvement need to be explored. A key limitation is that this is a single case. The paucity of other cases in the literature that also demonstrate effectiveness makes the evidence limited. Further studies with a larger number of patients, such as a case series or studies that compare the effectiveness of CRRT vs. TPE + CRRT, are required to definitively establish if TPE + CRRT is an effective treatment modality for acute ibuprofen poisoning.

## 4. Conclusions

While further investigation is warranted, this case report found that early TPE intervention may demonstrate much-improved ibuprofen removal than previously reported and that timing of the procedure may be an essential factor in its effectiveness.

## Figures and Tables

**Table 1:** Pre- and post-plasma exchange laboratory findings.

		Pre-TPE	Post-TPE	% Change
Hematology	WBC (4.5 10^9^/L–13.5 10^9^/L)	17.1	14.3	−16
	Hemoglobin (13–16 g/dL)	12.3	10.1	−18
	Hematocrit (37–49%)	37	30.3	−18
	Platelets (150 10^9^/L–400 10^9^/L)	181	133	−27
Coagulation	aPTT (26–36 s)	>200	>200	N/A
	PT (9.8–12.4 s)	29.5	24.2	−18
	INR (0.9–1.1)	2.7	2.2	−19
	Fibrinogen (200–400 mg/dL)	165	122	−26
	Heparin unfractionated (IU/mL)	0.8	0.5	−38
Blood acute toxins screen	Acetaminophen (10–20 μg/mL)	<10		
	Salicylate Acid (4–20 mg/dL)	<3		
	Alcohol (<10 mg/dL)	<10		
Urine drug panel	Amphetamine	Negative		
	Barbiturates	Negative		
	Benzodiazepines	Negative		
	Cocaine	Negative		
	Cannabinoid	Positive		
	Fentanyl	Negative		
	Methadone	Negative		
	Opiate	Negative		
	Oxycodone	Negative		
	PCP	Negative		

## Data Availability

The original contributions presented in this study are included in the article. Further inquiries can be directed to the corresponding author.

## Abbreviations

The following abbreviations are used in this manuscript:

TPE	Therapeutic Plasma Exchange
ASFA	American Society for Apheresis
Vd	Volume of distribution
CRRT	Continuous renal replacement therapy
AKI	Acute Kidney Injury
ECMO	Extracorporeal membrane oxygenation
